# Young Man With Suspected Foreign Body Ingestion

**DOI:** 10.5811/cpcem.2019.8.44080

**Published:** 2019-09-30

**Authors:** Erin F. Shufflebarger, Matthew C. DeLaney, David C. Pigott

**Affiliations:** University of Alabama at Birmingham, Department of Emergency Medicine, Birmingham, Alabama

## Abstract

As United States emergency departments (ED) and hospitals continue to contend with increasing numbers of patients presenting with complications of substance abuse, emergency physicians should also be aware of patients who may be smuggling illicit drugs. We report the case of a 26-year-old man who was transported to the ED for suspected drug smuggling. Abdominal computed tomography was notable for the presence of multiple tubular foreign bodies throughout the colon that were later identified as packets containing heroin. Body-packing patients present a high-risk clinical scenario that may result in massive, inadvertent drug exposure.

## CASE PRESENTATION

**Image 1:**
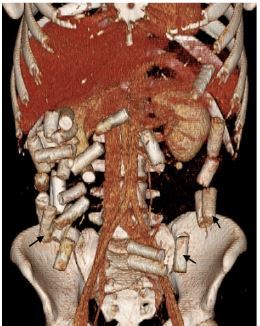
Computed tomography of the abdomen using three-dimensional reconstruction showing extensive tubular foreign bodies present throughout the colon (arrows).

A 26-year-old man was transported from the airport to the emergency department (ED) in police custody after being detained for suspected drug smuggling. The patient had no medical complaints and denied any ingestions. On arrival, he appeared anxious but readily consented to ED evaluation and treatment. He had normal vital signs and an unremarkable physical examination. His abdomen was soft, nontender, and nondistended without palpable mass. No signs of a recognizable toxidrome were present. We obtained abdominal computed tomography (CT) with oral and intravenous contrast for further evaluation (Image 1).

## DIAGNOSIS

### Body Packing

**Image 2:**
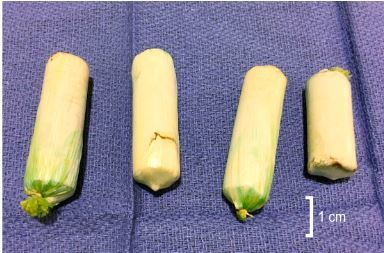
Multiple tubular, plastic-wrapped packets of heroin subsequently passed by the patient.

Abdominal CT revealed multiple radiopaque foreign bodies present throughout the colon without evidence of bowel obstruction. Whole bowel irrigation with four liters of oral polyethylene glycol was administered in the ED over approximately three hours. The patient was admitted to the intensive care unit (ICU) for observation, and over the following 24 hours he passed a total of 26 packets that contained heroin (Image 2). He was subsequently discharged in good condition in police custody.

“Body packing” refers to the ingestion of prepared packets of illicit drugs as a means of transport while avoiding detection.^1^ Providers must have a high degree of suspicion based on the available history as over 50% of patients will be asymptomatic on presentation.^2^ Abdominal radiographs have a specificity of 99% for packet detection, but their use is limited by reported sensitivities of 70%. Contrast CT imaging has a similar specificity but sensitivities of 99–100%.^3^ The patient should be monitored closely in the ICU for signs of systemic toxicity and obstruction, and treated with whole bowel irrigation until clear rectal effluent is achieved with passage of all packets. Although most patients have an unremarkable hospital course, in cases of packet rupture, mortality rates of over 50% have been reported.^4^ Management of symptomatic patients is based primarily on the type of substance ingested,

**CPC-EM Capsule**

What do we already know about this clinical entity?

*Available literature describes body packing as a means to transport illicit drugs and discusses the best imaging modality to detect these illicit substances*.

What is the major impact of the image(s)?

*This image is novel in its demonstration of 3-dimensional computed tomography reconstruction imaging used to detect extensive tubular drug packets throughout the colon*.

How might this improve emergency medicine practice?

*The recognition and diagnosis of body packing in asymptomatic patients is important, as missed ingestion could lead to potentially fatal complications*.
